# Therapeutically Targeting Cancers That Overexpress FOXC1: A Transcriptional Driver of Cell Plasticity, Partial EMT, and Cancer Metastasis

**DOI:** 10.3389/fonc.2021.721959

**Published:** 2021-09-03

**Authors:** Tania Ray, Terry Ryusaki, Partha S. Ray

**Affiliations:** ^1^R&D Division, Onconostic Technologies (OT), Inc., Champaign, IL, United States; ^2^3N Diagnostics Ltd., Belfast, United Kingdom

**Keywords:** Forkhead Box C1, transcriptional addiction, cancer stem cell, plasticity, epithelial-to-mesenchymal transition, metastasis, targeted therapy

## Abstract

Metastasis accounts for more than 90% of cancer related mortality, thus the most pressing need in the field of oncology today is the ability to accurately predict future onset of metastatic disease, ideally at the time of initial diagnosis. As opposed to current practice, what would be desirable is that prognostic, biomarker-based detection of metastatic propensity and heightened risk of cancer recurrence be performed long before overt metastasis has set in. Without such timely information it will be impossible to formulate a rational therapeutic treatment plan to favorably alter the trajectory of disease progression. In order to help inform rational selection of targeted therapeutics, any recurrence/metastasis risk prediction strategy must occur with the paired identification of novel prognostic biomarkers and their underlying molecular regulatory mechanisms that help drive cancer recurrence/metastasis (i.e. recurrence biomarkers). Traditional clinical factors alone (such as TNM staging criteria) are no longer adequately prognostic for this purpose in the current molecular era. FOXC1 is a pivotal transcription factor that has been functionally implicated to drive cancer metastasis and has been demonstrated to be an independent predictor of heightened metastatic risk, at the time of initial diagnosis. In this review, we present our viewpoints on the master regulatory role that FOXC1 plays in mediating cancer stem cell traits that include cellular plasticity, partial EMT, treatment resistance, cancer invasion and cancer migration during cancer progression and metastasis. We also highlight potential therapeutic strategies to target cancers that are, or have evolved to become, “transcriptionally addicted” to FOXC1. The potential role of FOXC1 expression status in predicting the efficacy of these identified therapeutic approaches merits evaluation in clinical trials.

## Introduction

Cancer screening strategies have proven to be successful in decreasing cancer-specific mortality in a variety of cancers (1–4). However, the clinical cure rates of patients who are diagnosed with cancer despite such screening measures still largely remain far from ideal (5). Our ability to potentially monitor cancer recurrence and metastasis events in real-time has now become possible with the development of various liquid biopsy modalities (circulating tumor cells, extracellular vesicles, cell-free DNA, etc.) (6). However, if we are to favorably impact current rates of cancer disease progression and exert meaningful decreases in cancer morbidity and mortality, we need to improve upon our ability to predict which newly-diagnosed patients are at the highest risk of suffering recurrence and metastasis events in the future. Biopsy-tissue derived molecular markers that predict heightened future metastasis risk still present the most pragmatic solution to this problem. This is because detection of such biomarkers utilizing standard immunohistochemistry (IHC) has a much higher likelihood of widespread global adoption owing to superior cost effectiveness and greater ease of integration into existing workflow of diagnostic pathology laboratories (7, 8).

Traditional clinical factors alone (such as TNM staging criteria) are no longer prognostically adequate for this purpose and we are in need of new prognostic biomarkers that are superior in their ability to predict cancer recurrence and metastasis risk (9). Absent such markers, our ability to pinpoint and identify which patients stand to derive the greatest clinical benefit of new therapeutic approaches being tested in clinical trials will be lost. As a result of not being able to “enrich” our clinical trial population with those patients who are most likely to derive survival benefit, we will ostensibly dilute the measured therapeutic efficacy in such trials, and may erroneously label a tested approach as being ineffective (10). Such biomarkers, once identified, may or may not play a functional or mechanistic role in driving the underlying aggressive biology contributing to the observed adverse outcomes. For this reason, recurrence/metastasis risk prediction needs to be performed in conjunction with the identification of the pivotal underlying molecular drivers responsible for increasing the probability of suffering a recurrence/metastasis event. This would allow formulation of a rational therapeutic strategy, based on targeting the identified underlying molecular driver mechanism, to ultimately derive clinical benefit.

**Figure 1:**
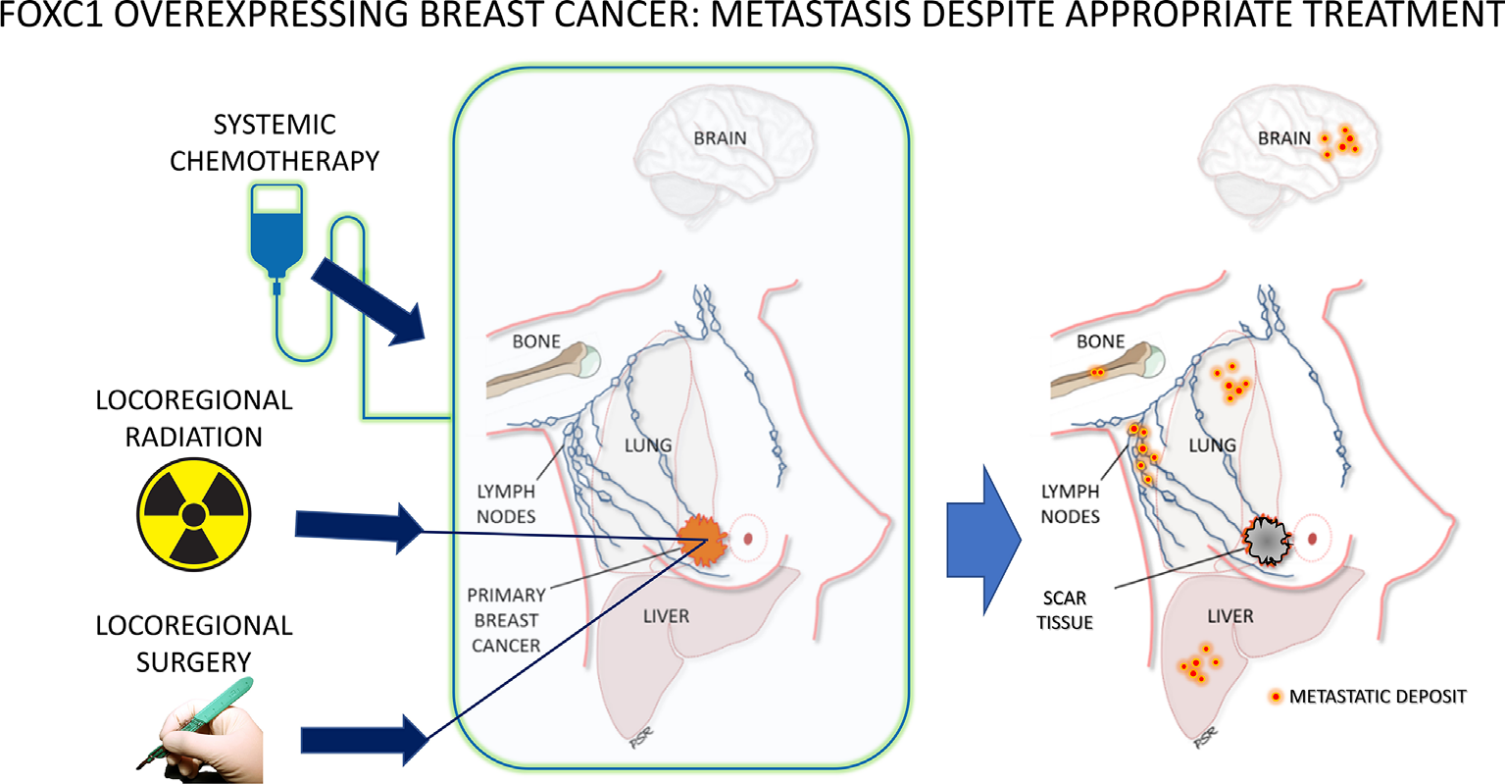
Clinical hallmarks of FOXC1-overexpressing breast cancer – metastatic recurrence despite adequate surgical resection, lymph node assessment and removal, radiation therapy and chemotherapy. For a biomarker to be associated with such aggressive behavior, resistant to common therapeutic treatment approaches, argues strongly in favor of this being a manifestation of the cancer stem cell phenotype.

In recent years, several excellent review articles on the role of the Forkhead box C1 (FOXC1) Transcription Factor (TF) in cancer have been published, highlighting the increasing recognition of its importance as a clinically useful biomarker and potential therapeutic target (11–15). The focus of this review is to summarize and draw inferences from the body of literature that implicates FOXC1 as a transcriptional driver of cancer progression and metastasis (**Figure 1)**. Herein we discuss the current state of evidence that supports the argument that FOXC1 plays this role by virtue of being essential for the emergence, maintenance and proliferation of cancer stem cells (CSCs). We present evidence of how FOXC1 plays a functionally important role in mediating a wide variety of cancer traits all of which are, essentially, cancer stem cell traits. Such traits include cell proliferation, cell plasticity, partial EMT, cell migration, cell invasion, chemoresistance and radio-resistance. In this review we present our viewpoints on the pivotal role that FOXC1 plays in this regard. FOXC1 dependencies develop as a consequence of dysregulated programs in cancer cells and affect clinical progression, therapeutic responsiveness and outcome often emerging to a level of dependence referred to as “transcriptional addiction.” Collectively, the elucidated mechanisms by which FOXC1 modulates aggressive cancer cell traits support our contention that FOXC1 is predominantly a transcriptional driver of cellular plasticity and a cancer stem cell phenotype. In this review, we also highlight several potential targeted therapeutic strategies, based on utilizing already available FDA-approved oral anticancer drugs, that may help achieve improved clinical outcomes for patients diagnosed with FOXC1-overexpressing pro-metastatic cancers. The therapeutic strategies described herein are potentially practice-changing in the field of oncology, and merit being tested in biomarker-driven, adaptive clinical trials.

## FOXC1: A Prognostic Biomarker of Cancer Progression and Metastasis

The earliest reports on the biologic role and function of the FOXC1 TF implicated it in abnormal pathologic conditions like glaucoma (16–23), congenital hydrocephalus (24), congenital renal defects (25, 26), congenital heart defects (21, 27–29) and Axenfeld-Rieger Syndrome (21, 30, 31), a congenital disorder characterized by glaucoma and congenital heart defects. Contemporaneously, several reports detailing the role of FOXC1 in normal physiology were also reported with regard to development of the cornea and the anterior chamber of the eye (32), renal development (33, 34) and cardiovascular development (27, 35). Also elucidated was its role in coordinating the embryonic process of primitive mesoderm cell fate (36) and migration of embryonic tissues (37).

The functional relevance and prognostic significance of FOXC1 in cancer was first reported in breast cancer in 2010 (38). Since that time, we and others have established the pivotal role that FOXC1 plays in coordinating the aggressive biology underlying cancer progression and metastasis in multiple cancers (**Tables 1**, **2**). Studies supporting the role of FOXC1 as a powerful prognostic biomarker are summarized in **Table 1** and includes both “solid” as well as “liquid” cancers like acute myelogenous leukemia (AML). Indeed, an excellent and comprehensive systematic review and meta-analysis on the prognostic role of FOXC1 in cancer concluded that FOXC1 expression in cancer is indicative of poor survival outcome (126). In support of FOXC1 playing a role in cancer progression, another meta-analysis reported that FOXC1 is 23.8% more likely to be expressed in late-stage cancers as opposed to early-stage cancers (127). Below, we highlight those studies that demonstrate the role of FOXC1 in predicting an aggressive clinical course, specifically highlighting its role as a predictor of metastatic recurrence.

**Table 1:** Clinical evidence supporting the role of FOXC1 in cancer progression and metastasis.

Cancer Type	Sample Size	FOXC1 in Progression and Metastasis: Clinical Evidence	Outcome Measured	Univariate Analysis		Multivariate Analysis		Reference PMID Number
				Hazard ratio (CI)	p-value	Hazard ratio (CI)	p-value	
AML	458		OS	–	–	1.784(1.29-2.46)	<0.001	(39) 26373280
AML	452		OS	1.592(1.263-2.007)	0.0001	1.755(1.355-2.273)	<0.0001	(40)
	313	+	OS	1.539(1.208-1.961)	0.0002	1.678(1.280-2.201)	0.0001	
Breast	295		OS	–	0.0001	1.25(1.02–1.52)	0.02	(38) 20406990
	286	+	DMFS	–	<0.0001	–	–	
	232		OS	–	0.0476	–	–	
	122		OS	–	0.0098	–	–	
	159		OS	–	0.0047	–	–	
Breast	724		OS	3.364(1.758–6.438)	0.0002	3.389(1.928-7.645)	0.0001	(41) 21424368
Breast	1975	+	DSS	1.71(1.31 to 2.23)	<0.001	1.55(1.17 to 2.06)	0.003	(42) 26041837
Breast	1986	+	DSS	1.973(1.802-2.961)	<0.0001			(43) 26565916
Breast	120	+	DFS	2.62(1.05-6.50)	0.038	2.58(1.04-6.42)	0.041	(44) 28493031
Cervical	219	+	OS RFS	– –	– –	2.928(0.508-6.585) 2.776(0.207-7.538)	0.021 0.035	(45) 28386355
Cervical	76		OS		0.0094			(46) 29328284
Colon	363	+	OS DMFS	0.432(0.325−0.573) 0.422(0.319-0.558)	<0.001 <0.001	0.668(0.492−0.907) 0.617(0.457–0.834)	0.010 0.002	(47) 29884889
Colon	361 185	+	OS OS DFS	1.190(1.023-1.389) 3.371(1.745-6.513) 2.557(1.453-4.497)	0.025 <0.001 0.001			(48) 30171256
Colon	361 86 134		OS OS OS	1.20(1.03-1.40)	0.002 0.004 0.002			(49) 31650548
Esophageal (SCC)	82		OS	–	0.014	–	–	(50) 25031703
Esophageal (SCC)	147	+	OS DFS	– –	0.023 0.037	– –	– –	(51) 28861321
Gastric	120		OS	0.273(0.144–0.521)	<0.001	0.370(0.184–0.745)	0.005	(52) 24329718
Gastric	422		OS	1.58(1.15-2.15)	0.0038	–	–	(53) 33987183
Hepatic	406	+	OS RFS	0.587(0.453–0.760) 0.566(0.434–0.738)	<0.0001 <0.0001	0.641(0.491–0.837) 0.649(0.495–0.852)	0.001 0.002	(54) 22911555
Lung (NSCLC)	125		OS	1.324(0.657–2.175)	<0.001	1.328(0.625–2.021)	<0.001	(55) 23264086
Lung (LUAD) Lung (LUSC)	500 494		OS OS	– –	0.0484 0.0363	– –	– –	(56) 30548656
Lung (NSCLC)	105		OS	2.237(1.220-4.098)	0.009	1.988(1.022-3.860)	0.043	(57) 31597217
Melanoma	Stg III 139 Stg IV 169	+ +	DMFS DMFS	– –	<0.05 <0.05	– –		(58) 27533251
Pancreatic	85		OS	1.432(0.567-2.045)	<0.001	1.328(0.586-2.178)	<0.001	(59) 23242609
Tongue	92		OS	–	0.006	–	–	(60) 25130698

“-” indicates that information was not mentioned in the text or supplementary material of the specific publication, AML, acute myelogenous leukemia; CI, confidence interval; DFS, disease free survival; DMFS, distance metastasis free survival; DSS, disease specific survival; FOXC1, Forkhead box C1; LUAD, lung adenocarcinoma; LUS, lung squamous cell; NSCL, non-small cell lung cancer; OS, overall survival; RFS, recurrence free survival.

**Table 2:** Functional and mechanistic evidence supporting role of FOXC1 in plasticity, EMT, chemoresistance, cancer progression and metastasis.

Cancer Type	Transcriptional Addiction	Cell Proliferation	Cellular Plasticity/Stemness	EMT/pEMT/MET	Circulating Tumor Cells (CTCs)	Endocrine Resistance	Chemoresistance	Radioresistance	Cancer Progression/Metastasis	Molecular Pathway	Summary of Findings	Reference
Breast			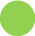	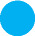							FOXC1 regulates CD44+ normal mammary progenitors	(61)
Breast		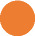	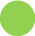	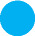					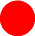		1^st^ Report of FOXC1 as a marker of aggressive traits	(38)
Breast							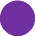				FOXC1 is associated with chemoresistance	(62)
Breast								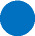			FOXC1 is associated with radioresistance	(63)
Breast	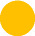			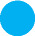			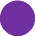			ERα	BRCA1 and GATA3 transcriptionally represses FOXC1	(64)
Breast				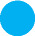	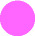					TGFβ	Elevated FOXC1 in breast CTCs	(65)
Breast									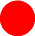		Transient vs Stable overexpression of FOXC1	(66)
Breast	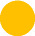								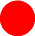	NFĸB	NFĸB inhibitor blocks FOXC1 mediated M/I	(67)
Breast				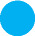					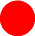	Raf/MEK/MAPK	FOXC1 downregulation associated with MET	(68)
Breast				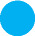	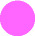				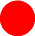	TGFβ	FOXC1 in breast CTCs associated with progression	(69)
Breast			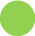								FOXC1 is enriched in mammary luminal progenitors	(70)
Breast	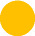								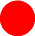	Ras/ERK, PI3K/AKT	EGFR inhibitor blocks FOXC1 mediated M/I	(71)
Breast							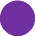				FOXC1 is a marker of neoadjuvant chemoresistance	(72)
Breast	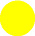	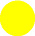							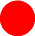	FOXCUT	FOXC1-FOXCUT form mRNA-lncRNA pair	(73)
Breast	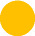		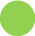							C-Wnt, NFĸB	NFĸB blocks stem cell escape following Wnt inhibition	(74)
Breast	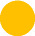		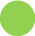	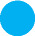					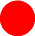		FOXC1/FOXA1 transcriptional balance in breast cancer	(74)
Breast			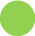				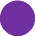			NC-Hedgehog	FOXC1 induces Gli2 activation and NC Hedgehog	(43)
Breast	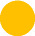									CDK7	CDK7-dependent SE-mediated FOXC1 addiction	(75)
Breast						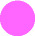				ERα	FOXC1 transcriptionally represses ERα	(76)
Breast									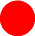		FOXC1 overexpression induces increased lung metastasis	(77)
Breast						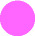				ERα	FOXC1 transcriptionally represses ERα	(78)
Breast							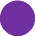				FOXC1 is a marker of adjuvant Anthracycline resistance	(44)
Breast										EGFR, NFĸB	NFĸB mediates EGF-induced FOXC1 expression	(79)
Breast				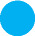					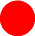	TGFβ, FGFR1	TGFβ upregulates FOXC1, triggers FGFR1 isoform switch	(80)
Breast			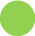								FOXC1 inhibits ELF5, lobuloalveolar development	(81)
Breast									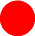	NC-Wnt, NFĸB	FOXC1 mediates Wnt5A-NfĸB-MMP7 induced invasion	(82)
Breast				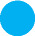							Alternative splicing switch in FLNB induces FOXC1, EMT	(83)
Breast			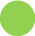						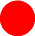	FAK-AKT-mTOR	ST8SIA1 regulates FOXC1	(84)
Breast	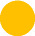								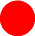	CXCR4	CXCR4-inhibitor blocks FOXC1 mediated M/I, metastasis	(85)
Breast							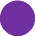			EZH2	EZH2 epigenetically represses FOXC1	(86)
Breast											FOXC1 upregulates LINC01123, ↓miR663a	(49)
Breast	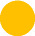		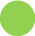	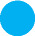					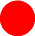	CDK7	FOXC1 contributes to CDK7-inhibitor sensitivity	(87)
Breast	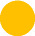		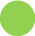						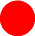		FOXC1 is most significant EMAT/metastasis activator	(88)
Breast	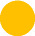		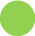						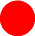	CDK7	FOXC1 is most significant invasion/metastasis activator	(89)
Prostate						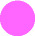			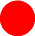		FOXC1 expression associated with AIPC progression	(90)
Prostate									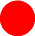	EGFR	EGFR upregulates FOXC1	(91)
Prostate	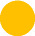	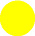							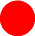		MiR-138-5P inhibits FOXC1-mediated M/I	(92)
Prostate	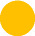	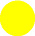							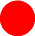		MiR-138-5P inhibits FOXC1-mediated M/I	(49)
Lung		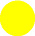		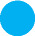					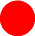		FOXC1 drives proliferation, EMT, invasion	(93)
Lung		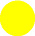		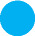					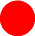	HIF1α	HIF1α transcriptionally upregulates FOXC1	(94)
Lung			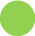				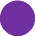			β-catenin, C-Wnt	FOXC1 transcriptionally upregulates β-catenin	(56)
Lung		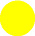							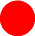		lncRNA CCAT2 upregulates FOXC1	(95)
Lung		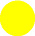							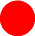	LOX	FOXC1 transcriptionally upregulates LOX, ↑metastasis	(57)
Lung		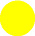							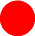	HIF1α	FOXC1 transcriptionally upregulates LINC00301, ↑HIF1α	(96)
Cervical				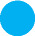					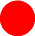	PI3K/AKT	FOXC1 mediates PI3K/AKT induced EMT, M/I	(45)
Cervical	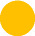								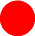		miR-374c-5p inhibits FOXC1-mediated M/I	(45)
Cervical								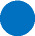	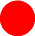		FOXC1 is associated with radioresistance	(29)
Cervical									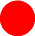	PI3K/AKT	FOXC1 mediates PI3K/AKT induced M/I	(46)
Endometrial		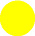							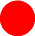		miR-204 inhibits FOXC1-mediated proliferation, M/I	(97)
Endometrial		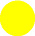							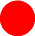		miR-495 inhibits FOXC1-mediated proliferation, M/I	(98)
Esophageal	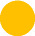									FOXCUT	FOXC1-FOXCUT form mRNA-lncRNA pair	(50)
Esophageal		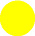	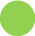	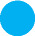							FOXC1 acts as transcriptional coactivator of, PBX1, ↑ZEB2	(51)
Gastric		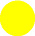								β-catenin, C-Wnt	FOXC1 transcriptionally downregulates DKK1, activating Wnt	(53)
Gastric		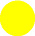							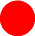	Wnt	FOXC1 transcriptionally upregulates GPX8, activating Wnt	(99)
Gastric		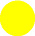	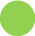						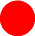		LINC00242 inhibits miR-141, upregulates FOXC1	(100)
Gastric		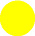					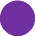		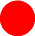		MCM3AP-AS1 inhibits miR-148, upregulates FOXC1	(101)
Gastric										EGFR	EGFR-FOXC1 upregulates histone H3C14	(102)
Pancreatic	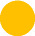	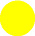	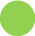								miR-138-5p inhibits FOXC1,↓ proliferation,↓ tumor growth	(103)
Pancreatic	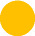	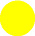		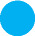					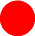	PI3K/AKT/mTOR	FOXC1 and IGF1R positively regulate each other	(104)
Hepatic	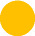	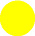		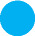					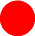	VEGF	FOXC1	(105)
Hepatic	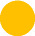			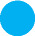					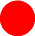	NEDD9	FOXC1 transcriptionally upregulates NEDD9, ↑metastasis	(54
Hepatic									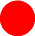	IL8,PI3K/AKT,CXCR1	IL8-dependent PI3K/AKT/HIF1α upregulates FOXC1	(106)
Hepatic									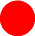	HOTAIR	FOXC1 upregulates lncRNA HOTAIR, ↓miRNA-1	(107)
Colon									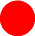	FGFR4	FOXC1 transcriptionally upregulates ITGA7, FGFR4	(47)
Colon		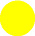	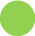								FOXC1 transcriptionally upregulates FBP1, ↑Warburg Effect	(48)
Colon							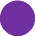				FOXC1 transcriptionally upregulates miR-31-5p, ↓LATS2	(108)
Colon	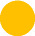								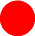		p38MAPK maintain FOXC1 protein stability, ↑metastasis	(49)
Colon							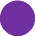		>		FOXC1 induces resistance to oxaliplatin, irinotecan	(109)
OSCC	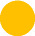								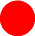	FOXCUT	FOXC1-FOXCUT form mRNA-lncRNA pair	(110)
OSCC	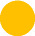	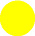							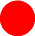		FOXC1 transcriptionally upregulates CCNB1/D1, MMP2/9	(47)
OSCC			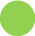				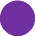		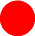		MCM3AP-AS1 inhibits miR-138, upregulates FOXC1	(111)
Tongue	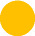	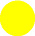							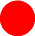	TGFβ	miR-639 inhibits FOXC1-mediated EMT, M/I	(60)
SACC	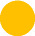	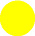		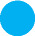					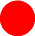		miR-582-5p inhibits FOXC1, ↓M/I, ↓metastasis	(78)
LSCC									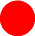		miR-204-5P inhibits FOXC1-mediated M/I	(112)
NP				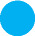							FOXC1 associated with low E-cadherin expression	(113)
NP	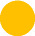			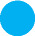					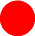		FOXC1 siRNA inhibited EMT, M/I	(114)
NP	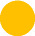			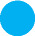					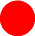		miR-4792 inhibits FOXC1-mediated EMT, M/I	(115)
NP	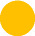			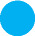						FOXCUT	FOXCUT siRNA inhibited FOXC1-mediated MMP7/9	(44)
OS	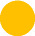			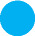					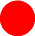		miR-133b inhibits FOXC1-mediated proliferation, M/I	(116)
OS	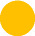	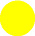							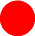	EZH2	FOXC1 transcriptionally upregulates EZH2	(117)
OS		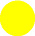							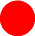	β-catenin, C-Wnt	Sp1/FOXC1/HOTTIP/LATS2/YAP/β-catenin cascade	(118)
OS	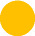	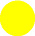							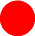		miR-185-5p inhibits FOXC1-mediated M/I	(119)
Melanoma			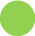						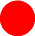	PI3K/AKT	FOXC1 mediates MST1R mediated colony formation, M/I	(120)
WT	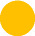	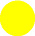							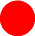		HOXB2 and FOXC1 synergistically drive progression	(121)
GB	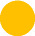								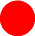		miR-133 inhibits FOXC1-mediated M/I	(47)
GB	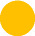			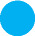					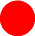	β-catenin, C-Wnt	FOXC1 siRNA inhibited EMT, M/I	(122)
Pituitary									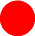		miR-133 inhibits FOXC1, ↓M/I	(120)
AML	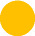	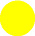	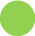								FOXC1, HOXA9 ↑clonogenic potential, ↓differentiation	(39)
T-ALL							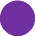			NC-Hedgehog	FOXC1 stabilizes GLI2, drives SMO-inhibitor resistance	(123)
HL	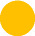	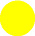								FGF2	OTX2 transcriptionally activates FOXC1	(124)
DLBCL	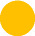								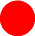		JUN upregulates FOXC1, drives lymphoma dissemination	(125)

AIPC, Androgen Insensitive Prostate Cancer; AML, Acute Myelogenous Leukemia; C-, Canonical; CTC, Circulating Tumor Cells; DLBCL, Diffuse Large B Cell Lymphoma; EMT, Epithelial-to-mesenchymal; GB, Glioblastoma; HL, Hodgkins Lymphoma; LSCC, Laryngeal Squamous Cell Carcinoma; MET, Mesenchymal-to-epithelial; NC-, Non-canonical; NP, Nasopharyngeal Carcinoma; OS, Osteosarcoma; OSCC, Oral Squamous Cell Carcinoma; pEMT, Partial Epithelial-to-mesenchymal; SACC, Salivary Squamous Cell Carcinoma; T-ALL, T Cell Acute Lymphocytic Leukemia; WT, Wilms tumor.

## Breast Cancer

The clinical evidence with regard to FOXC1 being a powerful prognostic indicator has been most extensively generated in breast cancer. FOXC1 mRNA and/or protein expression status has been demonstrated to be an independent, statistically significant, predictor of metastatic recurrence and poor survival. Multiple studies have now confirmed the clinical utility of FOXC1 expression, both at the mRNA as well as protein level, in predicting poor outcomes. Previous reports by us and others had shown that FOXC1 expression could accurately identify patients with the basal-like breast cancer (BLBC) molecular subtype, the most aggressive subtype of breast cancer (38, 41, 42, 66, 128–131). BLBC has been shown to exist as a “hidden” diagnosis, irrespective of receptor profile status, and is not restricted or confined to only triple negative breast cancers (TNBCs) (42). FOXC1* *expression status further correlated with increased incidence of brain and lung metastases and decreased metastasis-free survival in patients without lymph node involvement (38). More recently, in a study involving unbiased bioinformatic screening of transcription factors, FOXC1 was found to display the highest correlation with an epithelial-to-mesenchymal-to-amoeboid (EMAT) cluster having the worst associated disease-specific survival in lymph node negative patients (88). A clinical grade, quantitatively robust, immunohistochemistry-based molecular diagnostic assay has also been developed which measures FOXC1 protein in FFPE tissue and has a high degree of correlation with measurement of FOXC1 mRNA using qRT-PCR from matched FFPE tissue samples (42). The assay has undergone analytical and clinical validation, has achieved regulatory approval for breast cancer with the CE-mark designation and is available for *in vitro* diagnostic use in the clinic.

## Lung Cancer

The prognostic significance of FOXC1 in lung cancer was first reported in 2013 (55) and later confirmed by other independent investigators, both in lung adenocarcinoma as well as lung squamous cell carcinoma (56, 57). Studies have shown that high FOXC1 expression is more frequently associated with adverse clinical parameters and poor overall survival independent of other clinicopathological prognostic factors, including lymph node status. The data are clear on elevated FOXC1 expression being a predictor of lung cancer progression, but are not yet available with regard to predicting spread outside of the primary organ. It is important to note that in the case of lung cancer, twice as many patients ultimately succumb to respiratory failure than as a consequence of distant metastasis to sites outside the lung (132). Thus, lung cancer metastasis to locations or organs outside the lung does not appear to be the predominant cancer-related cause of death as in some other cancers that arise from the breast, colon, or skin (e.g. melanoma).

## Gastrointestinal Cancers

FOXC1 expression status has now also been shown to be a poor prognostic indicator in multiple gastrointestinal cancers including esophageal cancer, gastric cancer, liver cancer, pancreatic cancer and colon cancer. Two independent published reports provided initial insight into the potential prognostic significance of FOXC1 expression in esophageal squamous cell carcinoma with regard to overall survival. However, univariate and multivariate hazard ratio values were not available in the published reports (50, 51). One of these studies did however demonstrate that FOXC1 expression displayed a statistically significant association with disease-free survival as well (51). Similarly, two independent studies in gastric cancer thus far have provided preliminary confirmation of the prognostic significance of FOXC1 expression with regard to overall survival (52, 53). Additional independent validation studies performed with greater statistical rigor are therefore required to ascertain the true prognostic value of FOXC1 expression in the case of both esophageal as well as gastric cancer.

A fairly large investigation of patients diagnosed with hepatocellular carcinoma (HCC) demonstrated that FOXC1 expression was a powerful, statistically significant prognostic indicator of worse overall survival as well as recurrence free survival, independent of other clinical variables (54). This result was confirmed on both univariate as well as multivariate analysis. While this is a retrospective, single institution study, the sample size and level of statistical significance suggest that FOXC1 expression status will likely be of clinical value in predicting the prognosis of patients diagnosed with HCC. With regard to pancreatic cancer, there is a single report investigating the potential prognostic relevance of FOXC1 expression in pancreatic cancer (59). While the sample size was not large, the study was successful in demonstrating that FOXC1 is a statistically significant and independent prognostic indicator of adverse outcomes in terms of poor overall survival. This result was strengthened by the fact that it was valid on both univariate as well as multivariate analysis.

Amongst the gastrointestinal cancers, the evidence in support of the prognostic utility of FOXC1 is perhaps most robust in colon cancer. Three independent studies have confirmed the fact that FOXC1 expression status can in fact predict worse overall survival in colon cancer (47–49). One of these studies reports convincing data with regard to both overall survival and disease-free survival (48). Yet another provides further confirmation of FOXC1 being a statistically significant independent predictor of shortened distant-metastasis-free survival on both univariate as well as multivariate analysis (47). Thus, after breast cancer, clinical assessment of FOXC1 expression status for prognostic stratification of patients is most likely to be useful in colon cancer.

## Other Cancers

Within head and neck cancers, some functional and mechanistic studies have been published implicating FOXC1 in the biology of these tumors. However, investigations examining the prognostic role of FOXC1 are not yet available. A preliminary report on tongue cancer did demonstrate FOXC1 to be a statistically significant predictor of worse overall survival (60). However, the results of univariate and multivariate analysis were not accessible in the publication for review. Thus, information with regard to the prognostic role of FOXC1 in head and neck cancers is nascent at best, but does hold promise based on the findings of some initial studies on the functional significance of FOXC1. Studies investigating the prognostic significance of FOXC1 in nasopharyngeal carcinomas, laryngeal and oral squamous cell carcinomas are, therefore, needed. Similarly, with regard to melanoma, there is a single publication that reports FOXC1 to have prognostic significance in advanced Stage III or Stage IV melanoma in terms of distant metastasis-free survival. While highly insightful, the univariate and multivariate hazard ratios were not accessible in the publication for review. Thus, additional investigations are needed before we are able to draw any conclusions regarding the prognostic utility of FOXC1 expression in these cancers.

In contrast, data regarding the prognostic impact of FOXC1 expression in cervical cancer is more developed. Two independent studies have reported that FOXC1 possesses prognostic value with regard to predicting shorter overall survival (45, 46). More importantly, one of these confirmed FOXC1 to be a predictor of shorter recurrence-free survival on multivariate analysis (45). FOXC1 is a promising prognostic biomarker in cervical cancer and further studies should be performed to better delineate its clinical utility in this regard. In AML, high FOXC1 expression was found to be associated with adverse prognosis in comparison to low FOXC1 expression (39). Subsequently, high FOXC1 expression was significantly correlated with both refractoriness to induction chemotherapy as well as an increased risk of relapse (40). While further studies are needed in this regard, patients diagnosed with FOXC1+ AML should probably be recommended to enroll in clinical trials examining the efficacy of combination therapy protocols that combine various targeted therapies with standard induction chemotherapy. Such approaches may lead to their achieving minimal residual disease burden having no evidence of leukemic cancer stem cell markers, a known predictor of long-term remission following subsequent allogeneic bone marrow stem cell transplantation.

## FOXC1 Expression Associated With Good Prognosis

It is important to note, however, that in two very specific cancer types, elevated FOXC1 expression, in contradistinction to the above evident trend, proved to be a predictor of favorable prognosis. These departures from the norm are unexpected exceptions to the seemingly apparent rule that is supported by the overwhelming majority of research findings in support of FOXC1 expression being an adverse prognostic indicator. Thus, while the preponderance of evidence supports elevated expression of FOXC1 being an accurate predictor of adverse clinical outcomes, the two notable exceptions include ovarian cancer and Luminal B molecular subtype of breast cancer where elevated expression of FOXC1 predicts good prognosis.

In the case of ovarian cancer, a retrospective study (133) demonstrated that positive immunostaining for FOXC1 protein significantly decreased with advancing International Federation of Gynecology and Obstetrics Stage (I-II *vs.* III-IV) as well as pathologic subtypes from benign to borderline and malignant tumors trending towards good prognosis. However, it should be noted that the sample size was small for cystadenocarcinomas (n=40) and pathologic subtype (n=80). Although nuclear and cytoplasmic FOXC1 staining was observed in cell lines, the above conclusion was based solely upon cytoplasmic FOXC1 as FOXC1 was not detected in the nucleus of the ovarian tumors.

The second report on FOXC1 expression in Luminal B breast cancer (134) looks at FOXC1 as being predictive of favorable outcome and establishes the use of EZH2 methyltransferase inhibitors as a strategy to block metastasis. Specifically higher expression of FOXC1 was associated with increased relapse-free survival in Luminal B patients (HR=0.68 p=0.001), but not in BLBC patients (HR=1.01 p-0.94). This study demonstrated that FOXC1 activates certain anti-metastatic genes in the Luminal B breast cancer subset and that any pro-tumor effects of FOXC1 are overridden by the anti-metastatic functions of FOXC1 leading to an overall pro-survival effect in Luminal B patients expressing higher FOXC1 levels.

These highly exceptional findings merit further investigation into the context-specific subcellular localization of FOXC1, and factors that control and determine its post-translational stability and degradation. While initial insight into this aspect of FOXC1 regulation was provided in two published investigations of FOXC1 protein release, stability and degradation (83, 135), it remains to be established whether these or similar mechanisms may help explain the above observations related to FOXC1 expression in ovarian cancer and Luminal B breast cancer.

## FOXC1: A Functional Driver of Cancer Progression and Metastasis

**Figure 2:**
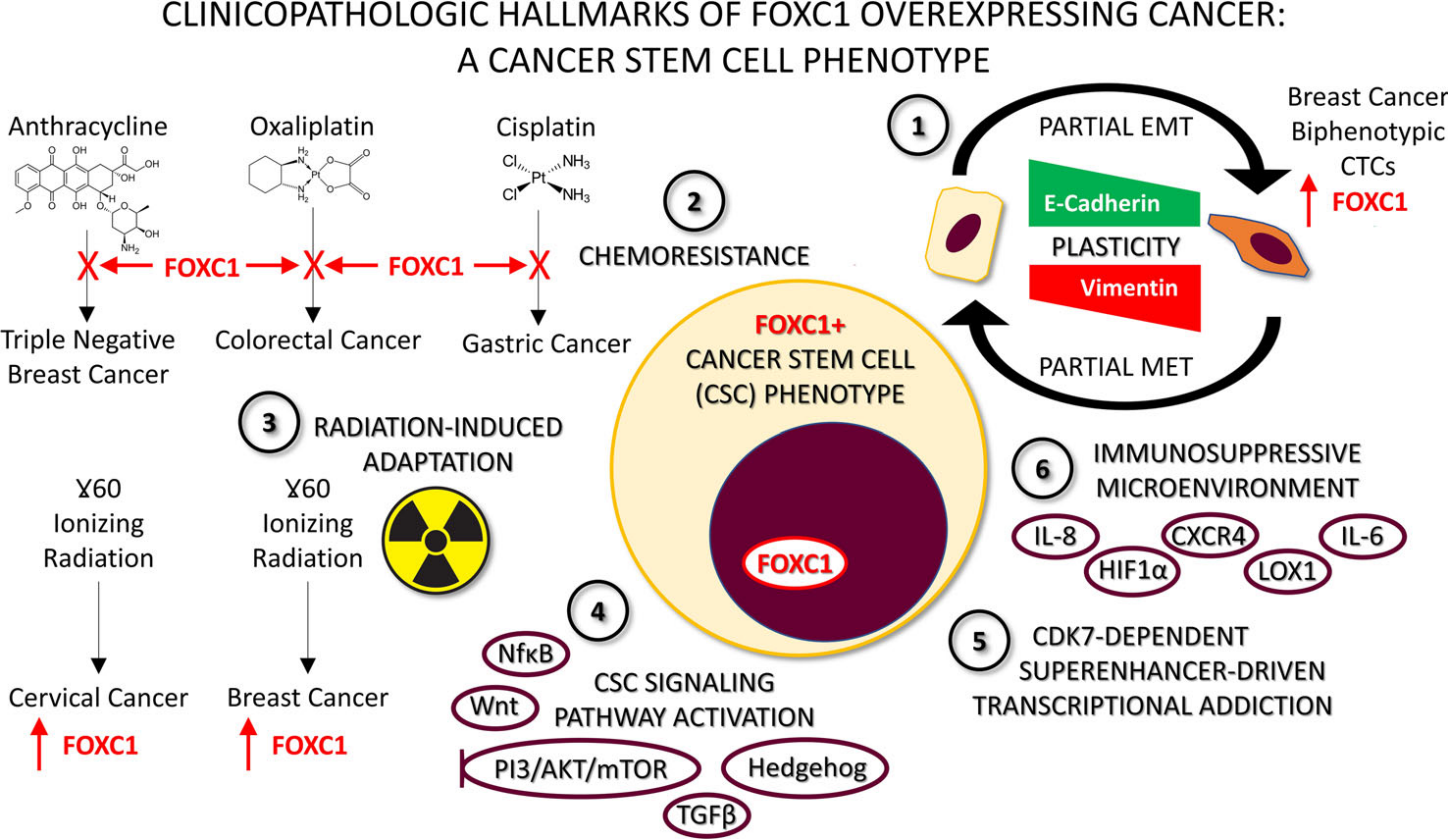
Characteristics of FOXC1-overexpressing pro-metastatic cancers. 1. Plasticity whereby FOXC1+ cells undergo partial Epithelial-to-Mesenchymal Transition (EMT), and can revert back by undergoing a partial Mesenchymal-to-Epithelial Transition (MET). Detection of FOXC1+ E/M hybrid biphenotypic Circulating Tumor Cells (CTCs) in the peripheral blood of breast cancer patients provides clinical evidence supporting the occurrence of this phenomenon *in vivo*. 2. Chemoresistance to a variety of chemotherapeutic agents driven by FOXC1 has been described in multiple cancer types with varied mechanisms. 3. Radiation-induced adaptation and subsequent resistance has been described in two different types of cancer cell line models to be characterized by FOXC1 overexpression. 4. Stem cell pathway activation, particularly of the non-canonical variety has been described for NFĸB, Wnt, Hedgehog, PI3K/AKT/mTOR and TGFβ signaling pathways in cancer. Moreover, they all converge on FOXC1. This has led to the suggestion that combination therapy with two or more pathway inhibitor drugs may be necessary to block FOXC1-driven cancer metastasis. 5. Superenhancer-driven transcriptional addiction to FOXC1 mediated by CDK7 has been described in breast cancer and was shown to be effectively thwarted using a CDK7 inhibitor drug. 6. FOXC1 contributes to an immunosuppressive microenvironment by upregulating multiple immunosuppressive factors including HIF1α, CXCR4, CXCR1 and LOX1. This helps explain how the cancer stem cell phenotype helps evade immune detection, and how FOXC1+ cancers are a valid target for immunotherapy approaches like immune checkpoint inhibitors.

The insight into FOXC1 as a prognostic predictor of cancer progression and metastasis was accompanied by a growing body of work that also demonstrated its functional importance as a molecular driver of these processes utilizing both *in vitro* and *in vivo* models **Figure 2**. The literature supporting this is summarized in **Table 2**. Several other cancer types than are not featured in **Table 2** have also been reported in which significant associations between the malignant phenotype and FOXC1 overexpression have been demonstrated. However, prognostic or functional roles of FOXC1 have yet to be elucidated in these cancers. These include carcinomas of the thyroid (136, 137), gallbladder (138), kidney (139, 140), non-melanoma skin cancer (141) and synovial sarcoma (142). Further investigations are required to explore the potential prognostic and functional significance of these initial reports.

By virtue of being a transcription factor, and being a central hub gene controlling a network of hundreds of genes, it is not surprising that upregulation of FOXC1 in cancer does cast wide influence on a number of biologic processes critical for tumor survival and propagation. While this includes proliferation as reported in a number of studies, what has been a hallmark feature of FOXC1+ status is that it appears to predominantly be responsible for a pro-metastatic phenotype. This is evident in **Table 2** based on the number of studies across a wide variety of cancers where this has been demonstrated to be the case. In addition to the clinical correlative studies outlined above, clear examples of the critical importance of FOXC1 in metastagenesis includes studies performed in breast cancer (77, 85), lung cancer (57), HCC (54, 143), colon cancer (49) and salivary gland cancer (144). In these studies, overexpression led to the development of an increased number of metastatic lesions in preclinical animal models. Conversely, siRNA-mediated knockdown of FOXC1 practically abolished metastatic propensity (49, 54, 85) in most of these models, confirming the critical dependence of these cancers on FOXC1 to drive the metastatic program.

Several molecular mechanisms have also been elucidated in this regard. These include induction of partial EMT that promotes enhanced cancer cell migration (discussed below), increased production of matrix metalloproteinases that promotes cancer cell invasion (**Table 2**), and interaction with other cell types in the tumor microenvironment by way of molecular crosstalk that further supplements and enhances these aggressive cancer cell characteristics (discussed below). Crosstalk with other cell types also leads to a state of pervasive immune suppression in the TME that significantly assists with cancer immune evasion. This also aids and abets the cancer cell in being able to continue on its aggressive pro-metastatic course. Cell crosstalk mechanisms pertinent to FOXC1+ cancers are discussed in more detail in a separate section below.

## FOXC1: A Driver of Aberrant Cellular Plasticity and Partial EMT

An epithelial-mesenchymal transition (EMT) enables cancer cells to depart from the primary tumor, invade surrounding tissue, and disseminate to distant organs. Several excellent reviews have implicated FOXC1’s role in EMT. After multiple initial reports that linked FOXC1 with EMT in various cancers, some recent reports have further refined our understanding that FOXC1 is in fact associated with a partial EMT phenotype comprising of hybrid E/M cells (**Figure 2**). Here we briefly summarize the literature in this regard.

Maheswaran and colleagues had found that mesenchymal cells expressing known EMT regulators, including TGF-β pathway components and the FOXC1 transcription factor, were highly enriched in circulating tumor cells (CTCs) and these mesenchymal CTCs were associated with disease progression (69). Similarly, Agelaki and colleagues found that EMT markers (Twist and Vimentin) are expressed in CTCs of patients with metastatic breast cancer (145). Additionally, Maheswaran and colleagues also noticed small populations of CTCs that were positive for both epithelial and mesenchymal markers by RNA-*in situ* hybridization, and these hybrid E/M CTCs were often enriched in patients with progressive disease after chemotherapy (69). In this same study, an index patient demonstrated dynamic switching between mesenchymal and epithelial CTCs upon each cycle of therapy, suggesting that CTCs maintain dynamic E/M plasticity (69, 146). This data supports the result from a study by Gupta and colleagues which utilized a DNA barcoding approach in the human breast cancer cell line MDA-MB-157 whereby a distinct clonal population of tumor cells was observed to fluctuate between epithelial and mesenchymal states, demonstrating intrinsic E/M plasticity (147). Additionally, they further demonstrated that a single clonal population of tumor cells maintained stable co-expression of both epithelial-to-mesenchymal markers, suggesting the fact, that it is possible for cells to be plastic enough to maintain both epithelial and mesenchymal characteristics.

In an independent study performed by Blanpain and colleagues that utilized a model of squamous cell carcinoma, the clonal population of tumor cells which maintain a hybrid E/M state, has been demonstrated to possess greater metastatic potential than either complete E polarized or complete M polarized cancer cells (148). In an HCC study, elevated FOXC1 expression was associated with enhanced trans-endothelial migration and microvascular invasion (105). However, siRNA-mediated knockdown of FOXC1 was only able to exert an incomplete reversion of EMT, characterized by decreased expression of mesenchymal markers (Vimentin, N-cadherin), but without an accompanying increase in a key epithelial marker (E-cadherin). Epithelial traits were only partially impacted in this condition, and E-cadherin remained unchanged in both expression level and distribution. In summary, this study provides evidence that metastasis may be more dependent on cells maintaining a high FOXC1 expression, which drives a partial EMT (having more of hybrid E/M characteristics) than it is on cells undergoing a complete EMT. This pool of highly plastic cells is more likely to survive in the bloodstream and represents the primary pool of cells from which metastatic lesions are seeded and arise. As such CTC FOXC1+ expression status may in fact help define those CTCs which are Metastasis Initiating Cells (MICs) (149).

## FOXC1: Role in Cancer Cell Crosstalk with Other Cells in the Tumor Microenvironment

Tumor cells are surrounded by a heterogeneous and complex tumor microenvironment (TME). The TME consists of a tumor specific extracellular matrix, which recruits an abundance of non-cancer cells including epithelial cells, endothelial cells, mesenchymal cells, immune cells and fibroblasts, all of which interact with the primary tumor cell contributing to tumor progression and metastasis. TME-tumor signaling actively secretes chemokines, cytokines, growth factors, and other metabolites to create a dynamic changing environment (150, 151). A chronic inflammatory, pro-angiogenic and immunosuppressive environment is created through ECM remodeling and through TME-tumor crosstalk, cancer progression and resistance to therapy (152, 153). Infiltrating inflammatory cells can provide a chemotactic escape route for migrating cancer cells from the bulk tumor and modulate cell invasiveness (154, 155). Breast cancer cells and macrophages, through a reciprocal paracrine loop involving EGF, CSF-1, CSF-2 or CCL18, leads to EMT, increased cell motility, invasion and metastasis (156, 157).

Akin to the above general trend, crosstalk between FOXC1+ cancer cells and other cells of the TME has also been a prominently noted feature. Upregulated FOXC1 in tumor cells induces production and release of cytokines, chemokines and growth factors which mediates recruitment of stromal cells to the TME (**Figure 2**). Proinflammatory cytokine Interleukin-8 (IL-8) is a member of the CXC chemokine family of angiogenesis/inflammation-related chemokines, secreted by stromal (endothelial cells and fibroblasts) and tumor cells. All biological effects of IL-8 are mediated by 2 receptors designated as CXCR1 (IL-8RA) and CXCR2 (IL-8RB). IL-8 induces FOXC1 upregulation by activation of the PI3K/AKT pathway and Hypoxia Inducible Factor 1α (HIF1α) (106). Consequently, activated FOXC1 transactivates CXC chemokine receptor 1 (CXCR1), a crucial promoter of cancer cell motility through activation of Rho-GTPases (158), that increases invasion and metastasis in HCC (106). CCL2 (monocyte chemoattractant protein-1, MCP-1) is a potent chemokine for monocytes, and a variety of other immune cells, known to activate JAK2/STAT3 signaling (159). CCL2 was also transcriptionally upregulated by FOXC1 in a FOXC1 overexpressed HCC cell line, and appropriately repressed in a FOXC1 knockdown HCC cell line. This transactivation of CCL2 by FOXC1 significantly promoted macrophage infiltration and cancer metastasis in HCC mouse models. These findings were corroborated in human HCC tissues, where FOXC1 expression was found to correlate with levels of IL-8, CXCR1 and CCL2 expression, and infiltration of tumors by macrophages. What is important to note here is that while IL-8 induces FOXC1 transcriptional upregulation *via* a PI3K/AKT-HIF1α-driven mechanism, FOXC1 in turn transcriptionally upregulates the cognate receptor of IL-8 which is CXCR1, thereby establishing a self-sustaining positive feedback loop. With regard to a different cytokine, FOXC1 overexpression robustly increased NFκB-driven luciferase activity in breast cancer MDA-MB-231 and MCF-7 cells (67). Upregulated NFκB, in turn, induced interleukin-6 (IL-6) generation in MDA-MB-231 cells. The IL-6/STAT3/NFκB positive feedback loop is known to persistently activate breast stromal fibroblasts (160). Pharmacologic targeting of the IL-8/FOXC1/CXCR1 and FOXC1/NFκB/IL-6 positive feedback loops hold promise for deriving clinical therapeutic benefit in FOXC1+ pro-metastatic cancers. The chemokine CXCL12 and its cognate receptor CXCR4, a transcriptional target of FOXC1 was shown to play central roles in cancer proliferation, angiogenesis, invasion, tumor microenvironment, as well as drug resistance induced by chemotherapy (85). CXCL12 affects tumor cell biology *via* 1) direct stimulation of signaling pathways that promote cancer cell growth, metastasis, and angiogenesis; 2) indirect effects, including the recruitment of CXCR4/CXCR7-positive cancer cells to CXCL12-expressing organs.

FGFR1 is a proven transcriptional target of FOXC1 in breast cancer, following its own transcriptional upregulation by TGFβ pathway activation (80). FGFR1 inhibition is known to promote infiltration of myeloid-derived suppressor cells (MDSCs), that exhibit strong immunosuppressive activity, into the breast cancer TME and promote both cancer progression and metastasis. Inhibition of FGFR1 markedly diminished the level of MDSC infiltration (161) and efficiency of metastatic dissemination (113). Crosstalk between FGFR1 and macrophage derived chemokines CXCL1 and CXCL5 promote tumor formation and progression (162). FGFR1 also promotes the release of inflammatory chemokine CX3CL1 which recruits macrophages to the TME and promotes angiogenesis, both processes being effectively blocked upon treatment with a CX3CL1 inhibitor (163). FOXC1 also transcriptionally upregulates FGFR4 in colon cancer (47). FGFR4 upon ligation with FGF19 is known to promote drug resistance, cancer progression and metastasis. FGFR4 has also been demonstrated to be a poor prognostic indicator in colon cancer (164). The activated FGF19-FGFR4 pathway enhances GSK3β-βcatenin signaling, consequently inducing EMT and resulting in increased HCC metastasis (165, 166) and CRC metastasis (47).

## FOXC1: Interplay With Signal Transduction Pathways in Cancer

FOXC1 has been shown to play a critical role in the development and progression of multiple malignancies. Aberrant FOXC1 expression is involved in diverse tumorigenic processes, such as abnormal cell proliferation, cancer stem cell maintenance, cancer migration, and angiogenesis. However, although FOXC1 overexpression often drives aggressive traits in a wide array of human carcinomas, the mechanisms of FOXC1 deregulation that influence the oncogenic and metastagenic processes seem specific to each tumor setting. Here we present an overview of FOXC1 and its correlation with various signal transduction pathways that have been reported in various cancers and highlight how the signal transduction-FOXC1 connection may provide an effective modality to therapeutically intervene and block the aggressive progression of FOXC1+ cancers.

## FOXC1: Interconnected with EGFR, NF-κB, Ras/Raf/MEK/ERK, and PI3K/Akt/mTOR Signaling

FOXC1 and EGFR are both critical markers and functional regulators of BLBC. Cui and colleagues reported that EGFR activation regulates FOXC1 expression through ERK and AKT-mediated pathways in BLBC cells (71). In a separate study by the same group, NFκB transcription factor was found to regulate FOXC1 expression in BLBC cells through EGFR signaling (79). EGFR activation also promoted nuclear translocation of NFκB, which binds to the FOXC1 promoter. In the highly aggressive BLBC subtype of TNBC, FOXC1 regulated Pin1/NFκB signaling (67) (**Figure 2**). Further, FOXC1 overexpression in basal-like MDA-MB-231 breast cancer cells markedly induced phosphorylation of NFκB p65 subunit at Ser-546 and its translocation into the nucleus. Battula and coworkers discovered that ganglioside GD2 expression defined breast cancer stem cells (BCSCs) and ST8SIA1 regulated GD2 expression and breast cancer stem cell (BCSC) function by activation of the FAK-AKT-mTOR signaling pathway. They also showed that in primary TNBC, ST8SIA1 was highly expressed and its expression positively correlated with the expression of FOXC1 (84). In HCC cell lines, IL-8 activated expression of FOXC1 *via* the phosphoinositide 3-kinase/AKT signaling and HIF 1α. FOXC1 transcriptionally activated CXCR1 and CCL2, which promoted inflammation, invasion and metastasis (106). In cervical cancers and melanoma, it was shown that FOXC1 increased MST1R and activated the PI3K/AKT pathway to drive invasion and migration in melanoma cells (58). Overexpression of FOXC1 in MIA PaCa-2 Pancreatic Cancer cells resulted in increasing the active forms of AKT, PI3K, ERK, and p70s6k (104). Taken together, these studies supported the finding that therapeutic targeting of the EGFR/FOXC1/NFκB pathway and PI3K/AKT/mTOR in BLBC, PI3K/AKT/HIF-1α/FOXC1 axis in HCC and MST1R/PI3K/AKT in cervical cancers, melanoma and pancreatic cancers, may provide effective modalities for treatment.

## FOXC1 and Wnt Signaling

Both WNT5A and FOXC1 are up-regulated in TNBC cells and play a significant role in invasion and metastasis. Han and colleagues showed that FOXC1 binds directly to the promoter of WNT5A and up-regulates WNT5A expression in TNBC cells *via* NFκB signaling (82) (**Figure 2**). Increased WNT5A expression in TNBC cells is also associated with increase in MMP7 expression. Collectively, FOXC1-WNT5A-MMP7-NFκB signaling axis plays an important role in the migration, invasion, and distant metastasis of TNBC cells. Further, FOXC1 negatively regulates DKK1 (a WNT inhibitor) expression by binding to its promoter region, thereby activating Wnt pathway in gastric cancer cells. FOXC1 also forms a complex with unphosphorylated β-catenin protein in the cytoplasm thereby promoting the entry of β-catenin into the nucleus. Once inside the nucleus, it dissociates from β-catenin, thus regulating transcription of c-MYC, which promotes the proliferation of gastric cancer cells (53).

## FOXC1 and Non-Canonical Hedgehog Signaling

FOXC1 induces cancer stem cell (CSC) properties in BLBC cells *via* activation of Smoothened-independent Hedgehog (Hh) signaling (43) (**Figure 2**). This non-canonical activation of Hh signaling is specifically mediated by N-terminal domain of FOXC1 binding directly to Gli2, enhancing transcription-activating capacity of Gli2. Together with regulating non-canonical Hh signaling, FOXC1 overexpression also induces resistance to SMO-inhibitors targeting canonical Hedgehog signaling, thus further confirming that actions exerted by FOXC1 are in agreement with it being a marker of CSC function.

## FOXC1 and MAPK, IGF1/IGF1R, and LOX Signaling Axis

Aberrant expression of FOXC1 and activation of the FOXC1-p38-MAPK loop promotes tumor metastasis in colorectal cancer (CRC) (49). IGF-1R and FOXC1 regulate each other in pancreatic cancer and FOXC1 is a direct downstream signaling molecule of IGF-1/IGF-1R axis. IGF-1R upregulation in pancreatic cancer cells contributes to cancer progression and metastasis (104). A positive correlation between FOXC1 expression and lysyl oxidase (LOX) expression was established in NSCLC patient samples wherein FOXC1 activated LOX transcription to drive cancer progression through the FOXC1-LOX axis (57). These results suggest that FOXC1 has oncogenic properties that favor metastasis of various cancers.

## FOXC1 and TGF-Beta Signaling

Exogenous exposure to TGFβ1 increased FOXC1 mRNA during TGFβ1-induced EMT *via* Smad2 and Smad3 transcription factors. Hopkins and colleagues demonstrated that FOXC1 expression was activated during TGF-β1-mediated EMT events through the binding of Smad3 proteins to a region in the FOXC1 promoter, ~800 bp upstream of the transcription start site (80). In thus study, while FOXC1 was not essential for TGFβ-induced EMT, it was however critical for effecting an FGFR1 isoform switch that was critical for driving invasion and metastasis, that followed the TGFβ-induced EMT.

## FOXC1: Transcriptional Addiction in Triple Negative Breast Cancer

Transcription factors have been implicated in controlling extensive gene expression and regulating various cellular responses. Enhancers are regions of non-coding DNA, which mediate the transcription of adjacent genes, serving as a cis-regulatory element. Superenhancers (SEs) are a hyper active subset of enhancers, that recruit transcription factors, cofactors, and chromatin regulators to drive abundant expression of some significant genes (e.g., oncogenes) in the cancer context. FOXC1 is an SE-associated transcription factor that contributes to invasion, migration and metastasis in TNBC (75, 89). Young and colleagues had found that TNBC cells and ER-negative cells are exceptionally dependent on the expression of at least a subset of the active genes that are transcriptionally regulated by cyclin-dependent kinase 7 (CDK7) (**Figure 2**). Additionally, in this study it was demonstrated that TNBC cell proliferation is selectively sensitive to THZ1, a newly developed CDK7 inhibitor while ER and or PR-positive cells were largely unaffected by treatment of THZ1 (75). To seek potential biomarkers of THZ1 sensitivity, Tang and coworkers analyzed the mRNAs profile in breast cancer cells treated with THZ1 from the previous study and demonstrated that elevated expression of SOX9 was significantly associated with the sensitivity of THZ1 in TNBC (87). Furthermore, SOX9 and FOXC1 interacted with each other, to co-regulate the MYC signaling pathway in TNBC, while CDK7 inhibitor, THZ1 significantly disrupted the binding of SOX9 to FOXC1 and several enhancer and SE-associated transcription factors, increasing apoptotic cell death. In summary, these findings demonstrate that a collection of SE-associated TNBC genes (EGFR, FOSL1, FOXC1 and MYC) play a significant regulatory role in the proliferation, survival, invasion and metastasis of these CDK7-sensitive and TNBC-enriched cancers. As a logical conclusion, CDK7 inhibitors that block these targetable oncogenes could potentially serve as a rational targeted therapy option for patients diagnosed with TNBC.

## FOXC1: Regulator of Cancer Stem Cells, Quiescence and Tumor Escape Mechanism From Conventional Chemotherapy, Radiation Therapy and Targeted Therapy

FOXC1 plays an important role in mediating normal as well as cancer stem cell traits. In normal physiology, Yi and colleagues demonstrated that murine hair follicle stem cells induce FOXC1 to re-establish quiescence (167). In this study, FOXC1 was shown to help preserve quiescent stem cell identity by activating NFATC1 and BMP signaling. In an independent study, hair follicle stem cells were demonstrated to have significantly higher FOXC1 expression, where it helps govern their proliferation and conserves their tissue-regenerating potential compared to downstream, more differentiated hair follicle cells (168). FOXC1 also helps reprogram murine epidermal cells to induced functional sweat gland-like cells, thus proving its potential to determine sweat gland fate *in vitro* (169). In murine reticular cells, FOXC1 expression is essential for maintenance of the niche where adult hematopoietic stem cells reside (170). These finding were later corroborated in the human system as well (171). These studies collectively highlight the role FOXC1 as a key transcriptional regulator of normal stem cell activity.

Cancer stem cells (CSCs), are a small population of cancer cells that recapitulate most normal stem cell traits, but also play an essential role in tumor initiation, maintenance, progression, metastasis, drug resistance to anti-cancer drugs and metastatic recurrence. Recent studies have indicated that FOXC1, which is associated with a wide variety of cancers, is also strongly associated with mediating CSC activity (**Figure 2**). Cui and coworkers reported that FOXC1-overexpressed MDA-MB-231 cells, when injected orthotopically into the mammary glands of BALB/c nude mice, resulted in a marked increase in tumor formation efficiency compared to the control group (43). On the contrary, when FOXC1-knockdown BT549 cells were injected into the mouse mammary glands, tumorigenesis was completely inhibited. *In vitro*, FOXC1 overexpressed SUM159 and MDA-MB-468 cells showed enhanced aldehyde dehydrogenase activity, the increase of which is used for characterizing breast CSC. The above results indicate that FOXC1 positively regulates CSC properties of BLBC cells *in vivo* and *in vitro*. In NSCLC, Xu and colleagues demonstrated that FOXC1 knockdown reduced CD133+ cell percentage, suppressed self-renewal ability, decreased expression of stemness-related genes (OCT4, NANOG, SOX2 and ABCG2) and inhibited NSCLC cell tumorigenicity *in vivo* (56). Battula and coworkers discovered that ganglioside GD2 expression defines breast cancer stem cells (BCSCs) (84). In thus study, ST8SIA1, which regulates GD2 expression was found to be positively correlated with FOXC1 upregulation.

Chemotherapeutic drug resistance is a well-established cancer stem cell property. Mullan and colleagues were the first to show that FOXC1 acts as a mediator of drug resistance to a chemotherapeutic drug regimen comprising 5-fluorouracil/epirubicin/mitomycin C (FEM) as well as to docetaxel (64) (**Figure 2**). Basal levels of FOXC1 expression were increased in FEM-resistant clones compared to parental MDA-MB-468 cells. Additionally, knockdown of FOXC1 in MDA-MB-231 cells resulted in increased sensitivity to treatment with docetaxel. In NSCLC, FOXC1 knockdown increased cisplatin and docetaxel sensitivity and reduced gefitinib resistance, whereas FOXC1 overexpression enhanced CSC-like properties and resistance to cisplatin and docetaxel (56). Oxaliplatin (OXA) is currently used as first-line chemotherapy to treat stage III and stage IV metastatic colorectal cancer (CRC). Transcription factor FOXC1 binds to the miR-31 promoter to increase the expression of miR31-5p and regulate LATS2 expression in CRC, resulting in cancer cell resistance to OXA (107). LincRNA MCM3AP−AS1 induced upregulation of FOXC1 expression, indicating cisplatin resistance in gastric cancer patients (101). Xu and coworkers investigated the effects of FOXC1 on chemosensitivity in TNBC patients and found that only a minority of TNBC patients have an excellent outcome after receiving standard chemotherapy (44). Despite receiving standard cytotoxic chemotherapy, approximately 30–40% of patients with early-stage TNBC develop metastatic disease. They showed that a significant percentage of TNBC patients who had suboptimal outcomes with anthracycline-based standard chemotherapy were FOXC1 positive.

Therapeutic resistance in cancer also includes resistance to endocrine therapy with anti-estrogen drugs. In breast cancer, Cui and colleagues demonstrated that ectopic FOXC1 expression in ERα-positive MCF7 luminal breast cancer cells greatly diminished the effects of growth-stimulatory B-estradiol and growth-inhibitory antiestrogen treatment with Tamoxifen and Fulvestrant (76). Furthermore, in breast cancer patients with ER-positive primary tumors who received tamoxifen treatment, FOXC1 expression is associated with decreased or undetectable ER expression in recurrent tumors post endocrine treatment. In another independent study by Wang and coworkers, overexpression of FOXC1 decreased expression of ERα protein and reduced cellular responses to estradiol and tamoxifen, while knockdown of FOXC1 induced expression of ERα protein and improved cellular responses to estradiol and tamoxifen (78).

## FOXC1: Role in Metabolic Programming in Cancer

Metabolic reprogramming is another essential hallmark of cancer (172). Specific metabolic processes can be directly involved in the transformation process or biological processes that support tumor growth. FOXC1, which has been shown to play an important role in the development and progression of multiple malignancies, also plays a pivotal role in metabolism. Xia and colleagues reported that FOXC1 could inhibit the cysteine metabolism-related genes, cystathionine γ-lyase (CTH) through upregulation of *de novo* DNA methylase 3B (DNMT3B) expression, which resulted in the decrease of cysteine levels and increase reactive oxygen species (ROS) levels (143). In human HCC cells FOXC1 was in turn upregulated by ROS-ERK1/2-p-ELK1 signaling axis. This positive feedback loop of ROS-FOXC1-cysteine metabolism promotes liver cancer proliferation and metastasis, and this pathway may provide a prospective clinical treatment approach for HCC. Altered glycolysis metabolism is a well characterized signature of invasive cancers. Li and coworkers investigated the role of FOXC1 in regulating glycolysis in CRC cells and found that knockdown of FOXC1 expression in LoVo and RKO cells *in vitro* markedly reduced glucose uptake and lactate production, while ectopic expression of FOXC1 in HT29 and SW480 cells increased glucose consumption and lactate production. Further, FOXC1 promoted glycolysis and proliferation in CRC cells by inhibiting a key gluconeogenesis regulating enzyme, fructose-1,6-bisphosphatase 1 (FBP1) expression by binding directly to the promoter regions of the FBP1 gene and negatively regulating its transcription.

## Therapeutically Targeting FOXC1+ Prometastatic Cancers

It is evident from the above narrative that multiple signaling pathways converge upon and regulate the FOXC1 transcription factor. FOXC1, in turn, influences and coordinates multiple biological processes, again utilizing a variety of downstream signaling pathways, by which FOXC1 pro-metastatic cancers participate in the maturation of the aggressive migratory phenotype leading to metastasis. In some cases, self-sustaining positive-feedback loops are created which make their targeted interruption particularly attractive therapeutic strategies to test in the clinic. While further mechanistic elucidation of the upstream regulators and downstream mediators of FOXC1 activity are required, certain preliminary therapeutic strategies have begun to emerge based on the foundational investigations that have thus far been completed. Below we describe some of these potential strategies which hold promise and merit further consideration during the clinical trial design process.

## NFĸB Signal Transduction Pathway

**Figure 3:**
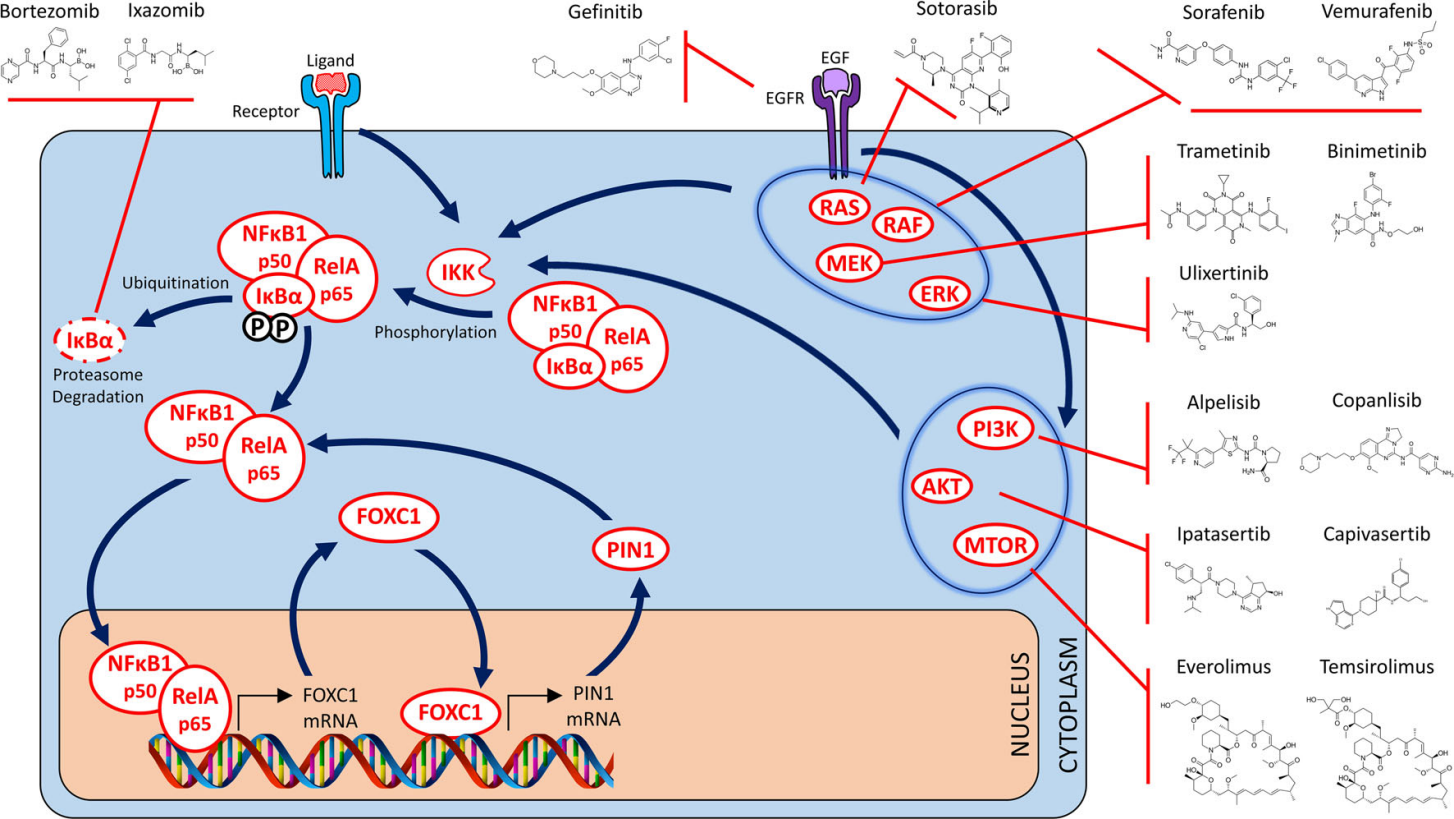
Targeted NFĸB, RAS/MAPK and PI3K/AKT/MTOR therapy strategies for FOXC1-overexpressing prometastatic cancers – I. Drugs that target the NFkB signaling pathway (Binding of transcriptional coactivator and RNA polymerase to p50/p65 heterodimer not shown). Indirect targeting opportunities are also depicted where targeted inhibition of alternate signaling pathways effectively blocks activation of the NFĸB signaling pathway as well. These include targeting of the EGFR receptor, various members of the RAS/MAPK signaling pathway (including RAS, RAF, MEK and ERK), as well as the individual members of the PI3K/AKT/mTOR signaling pathway.

As described above, the NFĸB signaling pathway and FOXC1 can in certain contexts reinforce one another’s actions indefinitely, thereby constituting a self-perpetuating positive feedback loop that contributes to the maintenance of cancer stem cell traits. This suggests that it is a potential therapeutic vulnerability that could potentially be exploited to improve survival outcomes (**Figure 3**). Significant preliminary data in support of such an approach being therapeutically actionable in the clinic was provided by a preclinical study (173). Utilizing a genome-wide siRNA screen, proteasome addiction was identified as a vulnerability of basal-like TNBC cells. Basal-like TNBC cell lines, known to have elevated FOXC1 expression status, were selectively sensitive to proteasome inhibitor drugs, proportionate to their relative level of FOXC1 expression. Proteasome inhibition effectively blocked tumor-initiating cell function *in vitro*, and significantly reduced tumor growth and metastatic dissemination to the lungs *in vivo*. Further evidence to support such an approach was obtained in another preclinical study wherein the Wnt signaling pathway (a CSC-associated pathway) was therapeutically inhibited in an *in vitro* FOXC1+ cancer model (74). While targeted inhibition of Wnt signaling was initially successful in markedly inhibiting mammosphere formation efficiency (a surrogate marker of cancer stem cell activity), resistant clones did emerge and mammosphere formation ability was regained. However, concurrent treatment with Bortezomib prevented the emergence of such resistant clones and effectively blocked the cancer stem cell escape mechanism.

Bortezomib and Ixazomib are two examples of FDA-approved drugs that are approved for use in the clinic to treat multiple myeloma. While Bortezomib is required to be administered intravenously (IV), Ixazomib is an oral drug with a superior toxicity profile, making it an ideal candidate for evaluating therapeutic efficacy against FOXC1+ pro-metastatic cancers by targeting the NFĸB signaling pathway *via* proteasome inhibition. Based on this rationale, a Phase I/II clinical trial (AGMT MBC-10) is currently underway to examine the efficacy of Ixazomib in combination with Carboplatin in previously treated advanced TNBC (174). Marizomib, a next generation IV/oral, brain-penetrant, proteasome inhibitor which also displays dual oxidative phosphorylation inhibitory action, has been shown to possess excellent efficacy in an *in vivo* preclinical study of TNBC utilizing both nude mouse/syngeneic animal models, as well as patient-derived xenografts (175). Marizomib not only caused a marked decline in tumor growth, it significantly reduced the development of lung and brain metastasis as well. Marizomib is currently being evaluated in multiple Phase I/II and III clinical trials in a variety of cancer types. Thus, FOXC1 expression status may serve as a companion/complementary diagnostic for the proteasome inhibitor class of drugs by virtue of their ability to disrupt the NFĸB-FOXC1 positive feedback loop.

## RAS/RAF/MEK/ERK/MAPK Signal Transduction Pathway

Utilizing a bioinformatic screening method, the breast cancer molecular subtype that was found to be most susceptible to treatment with small molecular MEK inhibitors was the basal-like subtype (176). The MAPK signal transduction cascade has been demonstrated to regulate and promote CSC traits in an *in vivo* mouse model of BLBC (177). Treatment with a MEK inhibitor in this model was successful in decreasing tumor growth. Pertinent to this review, RAS/MAPK pathway was demonstrated to regulate FOXC1 expression in breast cancer (68), suggesting that targeted inhibition of this pathway may offer therapeutic benefit in FOXC1+ pro-metastatic cancers (**Figure 3**). This and other preclinical evidence led to inhibitors of this pathway being tested as a rational treatment strategy for TNBC (178). Subsequently it was demonstrated that RAS/MAPK activation was associated with reduced tumor infiltrating lymphocytes in TNBC and likely contributed towards immune evasion (179). This suggested the possibility that efficacy of immune checkpoint blockade might be further improved if combined with MEK inhibitor therapy. Since activation of this pathway was found to be elevated in patients diagnosed with TNBC who had already been treated with anthracycline-based chemotherapy regimens, the RAS/MAPK pathway may be an attractive therapeutic target in the subset of patients who relapse or have refractory disease (180, 181). Emergence of resistance is a recognized phenomenon when undertaking targeted therapy of any signal transduction pathway in oncology, and the RAS/MAPK pathway proved to be no exception. Several groups reported a seemingly compensatory activation of the PI3K/AKT pathway in response to RAS/MAPK inhibitor therapy, and therapeutic response was obtained upon employing PI3K/AKT inhibitor therapy in animal models of RAS/MAPK inhibitor-resistance (182). This argued in favor of utilizing PI3K/AKT inhibitors to address therapeutic resistance to RAS/MAPK inhibitor therapy, or to undertaking combination therapy with inhibitors of both the RAS/MAPK and PI3K/AKT pathway (183). Another proposed approach to overcome therapeutic resistance encountered with MEK inhibitor therapy, or to achieve greater efficacy than could be achieved with MEK inhibitor therapy alone, was to undertake simultaneous blockade of multiple members of this signaling cascade. Thus, dual inhibition of both MEK and ERK (184) or of MEK and RAF (185), in preclinical models, proved to be successful in blocking the emergence of resistance as well as to overcome acquired resistance to MEK inhibitor therapy.

## PI3K/AKT/mTOR Signal Transduction Pathway

PI3K activating mutations and PTEN deactivating mutations are both known to increase and augment signal transduction through the PI3K/AKT/mTOR pathway. And both of these pathway activating mutations are known to occur with greater frequency in TNBC (186). Furthermore, with regard to pathway activation status itself, the PI3K/AKT/mTOR pathway is known to be significantly more upregulated in TNBC as compared to other receptor subtypes of breast cancer (187–189), and is known to contribute to both hormonal therapy resistance as well as chemotherapy resistance. Preclinical data was generated supporting the efficacy of PI3K/AKT/mTOR inhibitors in *in vitro* and *in vivo* models of TNBC (190). Based on these promising findings, a concerted effort was undertaken to examine the therapeutic efficacy of mTOR inhibitors in various clinical trials, in both ER+ breast cancer as well as TNBC. Everolimus was found to prolong progression free survival in patients diagnosed with ER+ breast cancer in the advanced/metastatic setting who had developed resistance to hormonal therapy (191). However, two separate Phase II trials failed to show any demonstrable efficacy in TNBC (192, 193). However, an appropriate and suitable companion diagnostic was never established to better guide therapy in either ER+ breast cancer or TNBC. Therefore, at the present time, there is no way to predict which patients diagnosed with ER+ breast cancer, are most likely to respond to treatment with this class of targeted therapeutics. It is also therefore not known whether a specific subset within the TNBC patients tested in trials, did actually derive some clinical benefit, again because no correlation was found between therapeutic efficacy and the candidate companion diagnostic markers tested thus far.

From the clinical trial experience with targeted inhibition of both the RAS/RAF/MEK/ERK/MAPK and the PI3K/AKT/mTOR signal transduction pathways, we have learned that the responses have been variable and not consistent. Furthermore, undertaking a dual inhibitory approach of two different nodes (i.e. MEK and ERK, MEK and RAF, PI3K and mTOR) or dual inhibition of both pathways is often fraught with severely limiting toxicity issues. One potential reason is that the bulk of the preclinical data that provided the initial rationale for these trials was obtained in models of BLBC. However, at the time of patient recruitment for these trials, the TNBC criterion was employed instead. As we have learned time and time again, the BLBC and TNBC definitions are definitely not synonymous and have a widely variable degree of overlap. The assumption that TNBC status is a close enough approximation and adequate surrogate equivalent of BLBC status is known to be incorrect. This might very well be an important factor contributing to potential miscalculation of the therapeutic efficacy in these trials as BLBC or high FOXC1+ status was not employed for patient enrichment, nor to assess potential companion diagnostic utility (**Figure 3**). A retrospective reassessment of the prospectively accrued patient samples from concluded trials on these lines may help to shed light on how trials evaluating the efficacy of this class of drugs might be better designed in the future.

## TGFβ Signal Transduction Pathway

**Figure 4:**
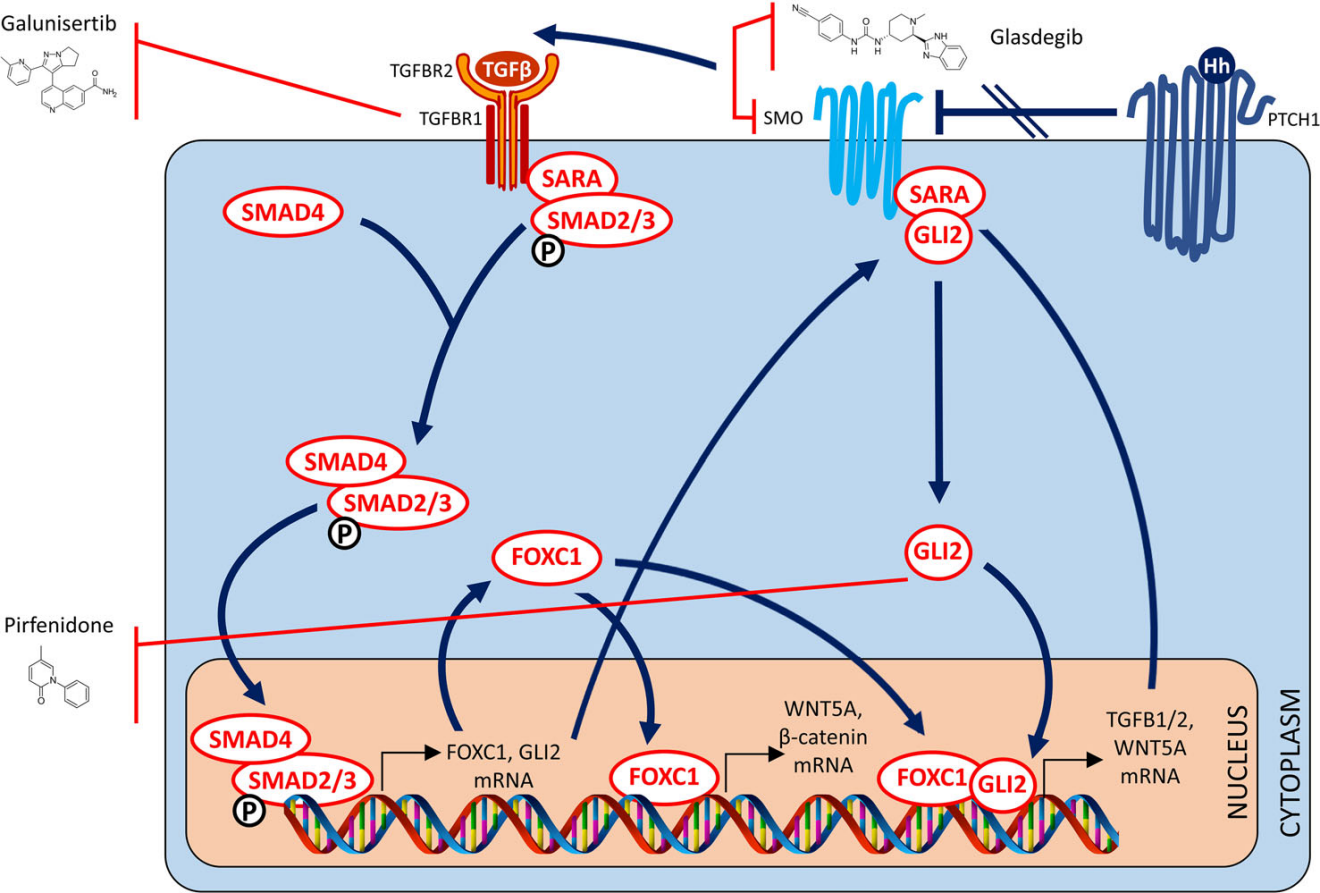
Targeted TGF-β and hedgehog therapy strategies for FOXC1-overexpressing pro-metastatic cancers – II. Drugs that target the TGF Beta and Hedgehog signaling pathways.

Targeted inhibition of the TGFβ pathway to target tumor growth, progression and metastasis has been controversial. This is primarily because activation of this pathway was demonstrated by two independent studies to variably exert anti-tumorigenic effects on the one hand, but pro-metastagenic effects on the other (194, 195). Interest was however, rekindled in utilizing this approach for therapeutic benefit, when it was demonstrated that TGFβ signaling, in certain subsets of cancer patients like BLBC, contribute to immunosuppression in the tumor microenvironment and immune evasion by the tumor. This was first suggested by a study in which the therapeutic effects of a TGFβR1 inhibitor being evident in a syngeneic, immunocompetent mouse model with an intact immune system, but not demonstrable in an athymic nude mouse model that lacks the ability to mount an immune response (196). Independent validation followed in another report wherein use of an anti-TGFβ antibody successfully suppressed metastasis mainly by inducing a highly significant enhancement of the CD8+ T-cell-mediated antitumor immune response (197). A subsequent independent study highlighted how such an approach held promise in the therapy of BLBC (198). Since then, multiple independent studies have reported that targeting TGFB with a therapeutic antibody (199), with a small molecule inhibitor (200) or with a bifunctional fusion protein (201) was able to overcome therapeutic resistance to immune checkpoint blockade, resulting in complete and durable therapeutic responses in otherwise poorly immunogenic tumors. Recently, another elegant and comprehensive study provided independent confirmation that therapeutic approaches based on TGFβ inhibition in breast cancer were most likely to be successful in patients with high grade, ER-negative disease of the claudin-low and basal subtypes (202). In summary, targeted inhibition of the TGFβ signaling pathway merits investigation in clinical trials to examine whether efficacy thereof can be predicted based on FOXC1 expression status (**Figure 4**).

## GLI2 Non-Canonical Hegdehog Signal Transduction Pathway

In a study by Cui and colleagues, FOXC1 was demonstrated to regulate CSC maintenance through activation of Smoothened-independent hedgehog signaling and binding to GLI2 in BLBC cells (43). Furthermore, FOXC1 over**-**expression also induced resistance to SMO-inhibitors targeting canonical Hedgehog signaling Therefore, blockade of the non-canonical pathway described above through targeted inactivation of Gli2 might be a potential strategy to overcome acquired resistance to canonical Hedgehog SMO-inhibitors, and attenuate FOXC1 induced tumorigenicity and metastatic dissemination. Glasdegib is an oral SMO-inhibitor which is FDA-approved for the treatment of newly-diagnosed AML in adults older than 75 years or those who have comorbidities that preclude use of intensive induction chemotherapy (203). In a preclinical study, Glasdegib was demonstrated to successfully target leukemia stem cells (204). Interestingly, although Glasdegib is a SMO-inhibitor, its effects downstream of its SMO-inhibitory action include GLI2 inhibition which abrogates leukemia stem cell dormancy (205). As such, efficacy of Glasdegib in AML and/or other relevant cancers may be predicted by FOXC1 expression status.

Perfenidone is an oral drug approved for treatment of Idiopathic Pulmonary Fibrosis (206, 207). Perfenidone has been proven to decrease fibrosis by decreasing TGFβ1 through a GLI2 inhibitory mechanism (208). However, by virtue of this same mechanism, Pirfenidone was recently demonstrated to be capable of inhibiting fibroblast activation and tumor-fibroblast crosstalk in the TME (209). Another study reported that treatment with Perfenidone was successful in reducing immunosuppressive capacity of cancer-associated fibroblasts in the TME (210). Most importantly, treatment with Pirfenidone was recently reported to augment the observed therapeutic benefit of PD1/PDL1 immune checkpoint blockade in syngeneic, immunocompetent mouse models of lung, liver and colon cancer (211). Collectively, these findings present a unique opportunity for testing in cancer clinical trials where FOXC1 expression status may be found to be useful in predicting efficacy of Perfenidone in ameliorating tumor growth and/or metastatic dissemination (**Figure 4**).

## CXCR1 Driven Cell-Cell Interaction

**Figure 5:**
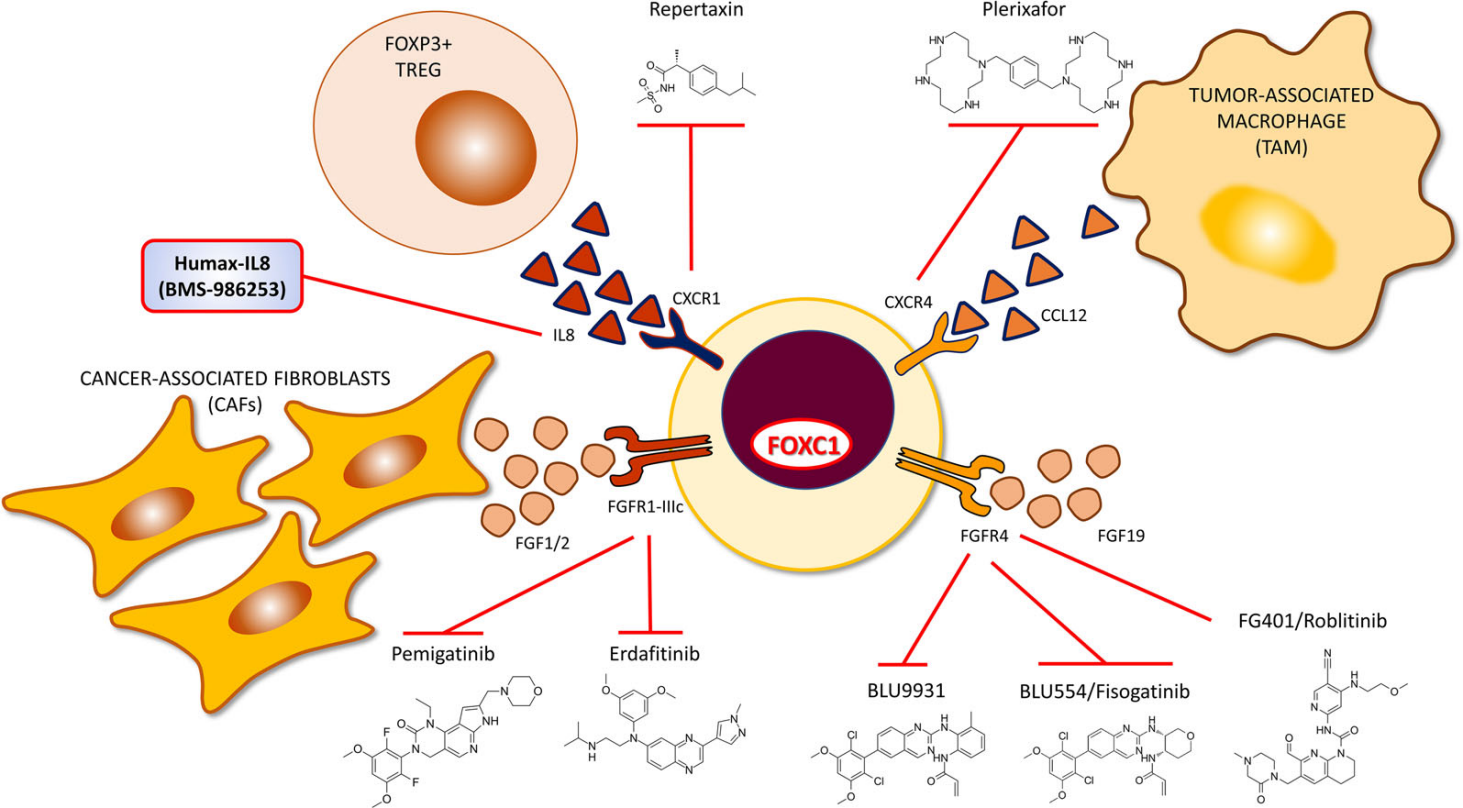
Targeted TME therapy strategies for targeting FOXC1-overexpressing pro-metastatic cancers – III. Drugs that target the IL8, CXCR1, CXCR4, FGFR1 and FGFR4 mediated cell-cell interactions in the tumor microenvironment.

FOXC1 transcriptionally upregulates CXCR1 in breast cancer (106). CXCR1 is a breast CSC marker (212), and upregulation has been reported to increase recruitment of FOXP3+ T regulatory cells to the TME and contribute to an immunosuppressive state (213). Repartaxin is a small molecule inhibitor of CXCR1 and has been demonstrated to have therapeutic efficacy in preclinical models of breast cancer (214) and gastric cancer (215). In another study utilizing preclinical models of HER2-positive breast cancer, Repartaxin successfully reduced breast CSC activity and demonstrated enhanced therapeutic efficacy when combined with Lapatinib (216). In a window of opportunity neoadjuvant trial, Repartaxin was successful in decreasing the breast CSC count in by more than 20% in preoperative tumors (217). Newer CXCR1 inhibitors have also been reported to have efficacy in preclinical models of RCC and HNSCC (218). Utilizing CXCR1 inhibitors to target CSCs is a promising therapeutic approach with sufficient pre-clinical data to merit examination of their therapeutic efficacy in FOXC1+ pro-metastatic cancers, especially in the setting of advanced/metastatic and/or recurrent cancers which are predicted to be enriched for FOXC1+ CSCs (**Figure 5**).

## CXCR4 Driven Cell-Cell Interaction

CXCR4 is a direct transcriptional target of FOXC1 in breast cancer that helps mediate increased invasion and metastasis in a preclinical model (85). In this model, treatment with Plerixafor, a CXCR4 antagonist was successful in reversing the CXCR4-mediated invasion and metastasis. Plerixafor is an oral inhibitor of CXCR4 currently FDA-approved as an HPSC mobilizer. The antitumor efficacy of Plerixafor in combination with a standard radio-chemotherapy protocol was evaluated in a murine model of cervical cancer (219). In another study, Plerixafor and another CXCR4 antagonist were observed to successfully reduce the incidence of bone metastasis in an animal model of prostate cancer (220). More recently, Plerixafor was found to mobilize cancer stem cells from their niche in both AML as well as glioblastoma (221). Beyond CXCR4 expression status itself, FOXC1 expression status may provide additional information for more accurately predicting the therapeutic efficacy of CXCR4 inhibitors in various cancers and merits examination in clinical trials (**Figure 5**).

## FGFR1 Signal Transduction Pathway

As discussed above, FGFR1 is a proven transcriptional target of FOXC1 in breast cancer. Erdafitinib is a pan-FGFR oral inhibitor currently FDA-approved for the treatment of advanced or metastatic urothelial carcinoma (222). Pemigatinib is another oral inhibitor of FGFR1/2/3 currently FDA-approved for the treatment of locally advanced or metastatic cholangiocarcinoma (223). FOXC1 expression status may help predict therapeutic efficacy and improve patient selection for treatment with this class of drugs (**Figure 5**).

## FGFR4 Signal Transduction Pathway

As earlier discussed, FOXC1 transcriptionally upregulates FGFR4 in colon cancer (47). Several selective small molecule inhibitors of FGFR4 have been developed (224–228) whose efficacy in HCC and various other cancers is currently being evaluated in early phase clinical trials. FGFR4 activation is closely associated with its specific ligand FGF19. Beyond FGF19 expression status, FOXC1 expression status may provide additional information for more accurately predicting the therapeutic efficacy of FGFR4 inhibitors (**Figure 5**).

## Conclusion

Over the course of the past decade, much has been learned regarding the important role played by the transcription factor FOXC1 in cancer. It is now well accepted that FOXC1 regulates a diverse set of biologically aggressive traits in cancer. Beyond studies of association, FOXC1 has been demonstrated to play a causative role in cancer stem cell biology that contributes not only to an aggressive phenotype but to an aggressive clinical course as well, often culminating in metastatic dissemination and death. From the first report of its central role in aggressive BLBCs more than 10 years ago, we have come to understand that FOXC1 plays a pivotal functional role in more than 16 different cancer types. This list is likely to keep growing. During this interval, our understanding has advanced significantly. From an ever-expanding understanding of the functional and mechanistic role of FOXC1 in cancer, we have also developed an appreciation of its potential application as a powerful prognostic biomarker. FOXC1 expression in cancer tissues, has been demonstrated to be capable of identifying those patients who are at heightened risk of suffering metastatic recurrence. This is of particular relevance in those patient subsets (e.g. lymph node negative patients) where, based on traditional clinical factors, a heightened recurrence risk would have been least suspected. The fact that FOXC1 expression can now be measured quite easily using an inexpensive, clinically validated and commercially available assay (42) means that testing can truly be accomplished on a global scale, even in resource-challenged settings. This is a huge step from a global oncology care perspective. By accurately identifying those patients with FOXC1+ pro-metastatic cancer who are at greatest risk, such a test serves to refine and improve resource utilization, to optimize delivery of life-saving chemotherapy and targeted therapy to the patients who need it the most. Testing to allow identification of metastasis risk early in the disease process is crucial in the fight to reduce glaring inequalities in global cancer control. Even by conservative estimates, a lack of such testing, if not remedied very quickly, will allow cancer to assume global pandemic proportions within the next decade (229).

While identification of elevated metastasis risk is important, it serves another purpose than the one delineated above. Not only would it allow for potentially life-extending systemic treatment (chemotherapy) to be administered to identified high risk patients, it would also allow for their targeted enrollment in the latest clinical trials. Both of these FOXC1 biomarker status-driven interventions have the potential to exert a significant decrease in overall cancer related morbidity and mortality. Beyond the above outlined points of clinical utility, however, sufficient evidence has now accrued to suggest that FOXC1 expression status might also find use to predict the efficacy of certain classes of targeted therapeutics in oncology practice. We have presented some such potentially therapeutic rationales in this review with the intention of spurring debate and stimulating discussion amongst the global oncology community. This of course will need many years of focused effort to first identify the most promising therapeutic approaches to target FOXC1+ pro-metastatic cancers, followed by carefully conducted clinical trials to test and prove their effectiveness. But if successful, such practice-changing advances hold the promise of diminishing the devastating prognostic impact of metastasis, currently the single largest contributor to cancer-related mortality, and extend the lives of cancer survivors.

## Author Contributions

PR and TaR conceptualized the study. PR, TaR and TeR performed critical literature review, wrote the manuscript, critically reviewed and edited the manuscript and were collectively responsible for all manuscript content.

## Conflict of Interest

PR, TaR, and TeR are employees of and have equity in Onconostic Technologies, Inc. and 3N Diagnostics, Ltd. PR is a named inventor on patents related to the role of FOXC1 in cancer.

## Publisher’s Note

All claims expressed in this article are solely those of the authors and do not necessarily represent those of their affiliated organizations, or those of the publisher, the editors and the reviewers. Any product that may be evaluated in this article, or claim that may be made by its manufacturer, is not guaranteed or endorsed by the publisher.
